# Line manager perspectives on workplace-based efforts to reduce sickness absence: a qualitative study

**DOI:** 10.1080/17482631.2025.2510560

**Published:** 2025-05-30

**Authors:** Lene Rasmussen, Maj Britt Dahl Nielsen, Anne Helene Garde, Jesper Kristiansen

**Affiliations:** a National Research Centre for the Working Environment, Copenhagen, Denmark; b National Institute of Public Health, University of Southern Denmark, Copenhagen, Denmark

**Keywords:** Absenteeism, sick leave, sickness absence intervention, workplace, line manager, normalization process theory

## Abstract

**Introduction:**

The purpose of this study is to examine line manager perspectives on two workplace-based efforts to reduce sickness absence (uniform procedures for managing sickness absence and initiating preventive actions), two components from an intervention to reduce sickness absence in public sector workplaces in Denmark.

**Methods:**

We performed 19 semi-structured interviews with line managers from four public sector workplaces. The interviews were analysed using thematic analysis (TA). Normalization Process Theory (NPT) was used as theoretical framework.

**Results:**

Uniform sickness absence procedures are meaningful and provide clear expectations for line managers and employees about roles and responsibilities during sick leave. Line managers expressed a desire for flexibility in adjusting the procedures to the individual needs of the employees. They also reported a need for proactive preventive actions that prevent sickness absence from occurring in the first place. The line managers reported lacking competencies to ensure appropriate sick leave management and that their own well-being was often overlooked.

**Conclusion:**

Future interventions should focus on improving the work environment instead of focusing solely on absenteeism. It is essential to consider the well-being of the line managers and provide adequate training, as this can affect their capability to ensure the well-being of, and reduce sickness absence, among their employees.

## Introduction

Sickness absence from work presents complex challenges with significant implications for society, workplaces, and individuals. At the societal level, it constitutes an economic burden, primarily due to expenditures related to salary payments or sickness benefits during periods of sick leave. In the public sector, high levels of sickness absence may compromise the quality and efficiency of welfare services, while simultaneously increasing work demands and stress among remaining employees, who may be required to accelerate their work pace or take on additional shifts to compensate for absent colleagues (Fukuda et al., 2018).

At the individual level, prolonged sickness absence is associated with a range of negative health outcomes. Studies have shown that extended time away from work can contribute to both physical and mental health deterioration, potentially increasing the risk of developing chronic conditions. In addition, sickness absence can lead to financial difficulties and social isolation, which may further exacerbate health problems and hinder the recovery process (Stöllman et al., 2025).

Given the wide-ranging consequences of sickness absence, there is a strong rationale for investing in preventive efforts. A recent systematic review underscores the importance of evidence-based strategies aimed at reducing sick leave, particularly those that address both physical and psychological aspects of health. By prioritizing the reduction of sickness absence, organizations can create healthier work environments, enhance productivity, and contribute to broader societal well-being (Bosma et al., 2025).

The workplace plays a complex role in sickness absence. Adverse psychosocial (Clausen et al., 2014; Margheritti et al., 2025) and physical (Andersen et al., 2016; Bláfoss et al., 2023) work conditions are associated with increased sickness absence. Moreover, individual characteristics (Čikeš et al., 2018), workplace culture (Ruhle & Süß, 2020), and work characteristics such as flexibility (Dellve et al., 2016) can influence employees absence behaviour. In the scientific literature, prevention of sickness absence in a workplace context includes primary, secondary, and tertiary prevention (Calvet et al., 2021). Primary prevention means efforts to reduce the risk of work-related sickness absence for all employees. It is aimed at risk factors in the work environment, e.g., stressful psychosocial or physical working conditions, and strengthening well-being, engagement, and job satisfaction. Secondary prevention focuses on employees who are at increased risk of sickness absence due to, for example, common mental disorders (CMDs) or musculoskeletal disorders (MSDs). The prevention effort involves reducing work-related risks for sickness absence and targeted measures, e.g., work adaptation, to avoid sickness absence and strengthen the employee’s capacity and ability to work. Tertiary prevention is the return to work (RTW) of sick-listed employees (Calvet et al., 2021). Since this level is not really *preventing* sickness absence but rather managing existing absenteeism, the term often used is *management* of sickness absence. Like primary and secondary prevention, sickness absence management involves reducing work-related risk factors for sickness absence and creating workplace conditions that enable a successful RTW. Consistent with this, this paper distinguishes between *prevention* of sickness absence and *management* of sickness absence. The former focuses on preventing sickness absence from occurring, for example by ensuring a healthy and safe work environment, while the latter focuses on managing existing sickness absence, such as maintaining ongoing contact with absent employees and supporting their RTW.

The line manager is central in the prevention and management of sickness absence (Preece, 2019). A review by Corbiere et al. on the role of stakeholders in the RTW of employees on sick leave due to CMDs highlights that “*The manager is recognised as having a pivotal role and plays an important role in the interface between upper management, rehabilitation, and health care providers, coworkers, and the injured worker”* (Corbière et al., 2020, p. 408). It is further argued that the roles and actions of the manager includes showing empathy and support during the process, respect the employee’s confidentiality (e.g., details on diagnosis or treatment), being open, showing flexibility and creativity regarding work accommodation to enhance RTW, communicating with other stakeholders and the absent employee, and ensuring respect within the team and towards the sick-listed employee, for example by handling negative or stigmatizing expressions about the employee or the work accommodations (Corbière et al., 2020). These roles and actions are also found in a review by Durand et al., which added that collaboration between the line manager and the absent worker is the basis for a successful RTW (Durand et al., 2014). These reviews focus on studies examining the role of the line manager in the RTW process of employee is already on sick leave, often due to CMDs or MSDs (tertiary prevention).

When it comes to line managers’ role in preventing absenteeism, i.e., preventing absenteeism from occurring or preventing recurrent short-term absenteeism from becoming long-term absenteeism (primary and secondary prevention), the literature is more sparse. A review by Kristman et al., found that lack of support from managers in implementing work accommodations can lead to work disability, thus arguing for the importance of training managers in developing and implementing these (Kristman et al., 2016). Another review by McDowell et al. also focuses on work accommodations for employees with mental health issues and emphasizes the importance of training managers, or at least ensuring access to information, on how to select and implement relevant workplace adjustments that meet employees’ needs (McDowell & Fossey, 2015).

The findings of the presented reviews show that some research on line managers’ role and actions regarding absenteeism has been done and that much focus has been on providing work accommodations. However, it is also evident that much of the research focus on tertiary prevention of sickness absence (RTW processes). There has been less research focus on secondary prevention, and we have not found research examining the role of line managers in preventing absenteeism across the entire workforce (primary prevention). Furthermore, research have focused on roles and actions of the line managers (Corbière et al., 2020; Durand et al., 2014), but only few studies have examined facilitators and barriers for carrying out the role, and these have also focus in tertiary prevention (Ladegaard et al., 2019; Stochkendahl et al., 2015).

Against this background, the aim of this study is to contribute to the existing research by examining line managers’ experiences regarding the prevention and management of sickness absence from a holistic perspective, i.e., with regard to primary, secondary, and tertiary prevention, as well as investigating the factors that influence their ability to fulfil their central role. We aim to use the findings to develop practical implications for more appropriate and effective prevention and management of sickness absence. This is done through the following research question: *How do line managers perceive sickness absence prevention and management in practice, and what insights can be drawn from these perspectives to enhance the effectiveness of these practices?*

## Materials and methods

### Research context

This study was conducted in the context of a complex intervention aiming to reduce sickness absence in public sector workplaces in Denmark. The intervention consisted of five core elements that must be implemented and integrated as part of the daily management of sickness absence. The five core elements include *data, sickness absence management, sickness absence prevention, sickness absence coordination, and organization*. The aim is that the core elements collectively provide a systematic approach to managing and preventing sickness absence in the workplace. The core elements of the intervention model are intended to be flexible, allowing workplaces to determine which initiatives to implement under each core element and how to operationalize them in practice. This means that the workplaces can adapt the intervention to the context in which it is implemented. The core elements are described in depth elsewhere (Rasmussen, 2024). Previous research has reported on *sickness absence coordination* (Rasmussen et al., 2024b) and *data* (Rasmussen et al., 2024a). Both studies found that it could be challenging for line managers to find time for handling sickness absence, including familiarizing themselves with and understanding data related to sickness absence and implementing preventive measures. This means that sickness absence management often is sidelined in favour of other managerial tasks, such as keeping daily operations running. In this paper, we focus on line manager perspectives on the two core elements: *sickness absence management* and *sickness absence prevention*. In short, *sickness absence management* includes clear and standardized organizational procedures on how to report sick from work, on the ongoing dialogue between the sick-listed employee and the workplace during sick leave (e.g., contact on day 1, day 5, and day 10), and when to invite an employee with excessive absenteeism to a sickness absence review meeting (such as being absent three times within six months). *Sickness absence prevention* includes implementing initiatives to strengthen the psychosocial and physical work environment, which could entail both primary, secondary, and tertiary prevention strategies (Rasmussen, 2024), meaning that the aim is to both prevent future sickness absence or stop absences from occurring in the first place. Together, the two core elements constitute specific measures or procedures that line managers must adhere to in their work to reduce sickness absence.

### Theoretical framework

To explore line managers’ perceptions on *sickness absence management* and *sickness absence prevention* in practice and how these insights can be used to enhance the effectiveness of these practices, we draw on Normalization Process Theory (NPT), an explanatory framework for the implementation, embedding, and integration of new practices. NPT presents four generative constructs that promote or inhibit routine embedding: coherence, cognitive participation, collective action, and reflexive monitoring (May & Finch, 2009; May et al., 2009). The constructs are presented in Table 1.

**Table 1.** The NPT constructs (as presented by May and Finch, 2009).

NPT construct	Description of construct
Coherence	How actors understand and make sense of a new practice. This includes whether the actors can differentiate the practice from previous practices, whether they attribute a positive value to the new practice, and whether they know what the new practice requires of them.
Cognitive participation	How actors actively engage themselves and others in driving the new practice. This includes aspects such as initiation and enrolment of key stakeholders.
Collective action	The work done to enact the new practice, and whether the practice is connected to the allocation of skills and resources within the organization and existing practices and rules.
Reflexive monitoring	How actors individually and/or collectively assess the new practice, formally and informally.

NPT focuses on agency, meaning the work actors do to enact a new practice. The theory has been used to examine the implementation of new practices within various settings (McEvoy et al., 2014). For instance, Holtrop et al. utilized NPT in their study to examine how care management became a routine practice in primary care (Holtrop et al., 2016), while Burau et al. applied NPT in their research investigating the implementation of a health promotion intervention in mental health services (Burau et al., 2018). Both studies found NPT helpful for examining implementation processes.

Examining the implementation process of the two core elements (*sickness absence management* and *sickness absence prevention*) is outside the scope of this study. However, the study is part of a larger research project aiming to examine the implementation of the intervention framework as a whole (all five core elements together). For this reason, we chose to use an implementation theory as theoretical framework for the overall research project. We chose NPT as a framework because of the theory’s focus on the integration of new practices. This is because the core elements of the intervention model must be integrated as part of an organization’s daily sickness absence management practice—that is, they must be normalized. In this study, this means that the two core elements must be integrated into daily practice at the participating workplaces. Therefore, NPT can help us shed light on which factors promote or hinder this process. The data collection section describes how NPT is applied in this study.

### Study design

The study is a qualitative study based on semi-structured interviews with 19 line managers from four public sector workplaces in Denmark that have been working on implementing the intervention framework over a two-year project period. The workplaces had received funding from a public fund to implement local workplace-based sickness absence interventions based on the framework. Eligibility criteria for funding included having high incidences of sickness absence or poor work environment (Rasmussen, 2024).

### Recruitment and participants

The workplaces were recruited in the fall of 2020 among 43 public sector workplaces that implemented the intervention framework. We used a purposeful sampling approach (Suri, 2011) to ensure that we recruited workplaces from all levels in the Danish public sector (municipal, regional, and governmental) to examine the operationalization of the core elements in different contexts within the public sector. We recruited one workplace from the governmental sector, one from the regional sector, and two from the municipal sector. The workplaces came from different work areas and included various occupations, such as nurses, healthcare workers, pedagogues, and prison officers, with the healthcare sector being the most represented. The sizes of the workplaces ranged between 600 and 4,500 employees, and each workplace consisted of several departments with at least one line manager. From each workplace, 2 to 8 departments were selected to participate in the data collection (20 departments in total). We contacted the project managers of the sickness absence interventions from each workplace, who appointed the departments and helped us contact the line managers. We emailed each line manager and informed them about the focus of the interview. One line manager did not respond to our invitation, resulting in 19 line managers participating in the data collection. We obtained verbal consent from all participants to participate.

All recruited line managers had staff responsibility and were responsible for managing sickness in practice, i.e., contacting sick-listed employees and following up on sickness absences. We interviewed 13 women and six men. The line managers came from departments of various sizes and differed in seniority, with some having many years of experience while a few were new to the role. This allowed us to include different perspectives in the study.

### Data collection

We collected the interviews from March 2021 to April 2022. Most interviews were held online or by telephone due to the COVID-19 pandemic (*n* = 16), while a few were held in person at the workplaces, when possible (*n* = 3). We developed a semi-structured interview guide with questions informed by NPT. For *sickness absence management*, we included questions about adherence to the sickness absence procedures, referring to whether the line managers follow the procedures and thus the operationalization of the procedures in practice (collective action). We also asked about line manager and employee attitudes towards the procedures, as these can influence both whether the line managers are committed to following the procedures (cognitive participation) and whether they follow them in practice (collective action). Lastly, we included questions regarding assessment of the procedures, referring to whether the line managers find the procedures meaningful, whether they achieve their purpose, and any suggestions for adjustments (reflexive monitoring). Examples of questions were: “Do you usually follow the sickness absence procedures you have in your organization? If not, why not?” and “Is there anything you think works well regarding the sickness absence procedures? Something that works less well or that you would change?”

For *sickness absence prevention*, the questions related to current preventive actions in the workplace and the operationalization of these, referring to how these preventive actions are implemented in practice (collective action), and assessment of the preventive actions, referring to whether they are working as planned and whether they are meaningful (reflexive monitoring). Examples of questions were: “Which preventive activities are you working with at [work unit]?” and “How are the activities implemented? What do you do to kick-start the activities in practice?”. Each interview had a duration of 45–60 minutes. All interviews were recorded and transcribed using the NVivo 12 software.

### Ethics

We obtained verbal consent from all participants before each interview, and the study follows the principles of the Declaration of Helsinki. To ensure the participants’ anonymity, all names, workplaces, and events have been deleted or changed in the transcripts.

### Data analysis

All interviews have been coded inductively using thematic analysis (TA) (Braun & Clarke, 2006). Following TA, we read the transcripts and developed initial codes based on the immediate patterns we identified across the collected data. The initial codes were discussed within the author group, including whether we had observed the same patterns and whether the initial codes were relevant to answering the research question. Overall agreement on the immediate patterns led to testing the initial codes on two interviews to determine if they could effectively analyse the data comprehensively. During this process, the codes were revised as necessary by rephrasing the code label to something more appropriate, deleting or adding codes, or merging multiple codes into one. We did this based on criteria such as whether there was enough data to support the respective code and whether the code was relevant to illuminating the research question. This process led us to a list of final semantic codes (Braun & Clarke, 2006), which were applied to all interviews. All interviews were coded by the first author and we developed a codebook to ensure that the interviews were coded consistently.

The next step typically involves comparing the final codes to organize them into themes that capture similarities in the codes (Braun & Clarke, 2006). As stated previously, we wanted to use the four NPT constructs as analytical framework. To do this, we organized the final codes under the respective NPT construct we assessed they related to, rather than developing our own themes. This decision was based on the assessment of the author group. For example, a code labelled “line managers” behaviours regarding procedures on sickness absence management’ was assessed to relate to the collective action construct, as the code encompassed data on how line managers follow the procedures in practice and, thus, the operationalization of the procedures. Another example is that a code labelled “suggestions for alterations” was put under “reflexive monitoring”, as this code referred to improvements suggested by the line managers to enhance a successful operationalization of a core element. Therefore, the four NPT constructs form the themes in the TA. Finally, we developed subthemes that capture the essence of the themes. These subthemes are formulated as promoting and inhibiting factors for the uptake of the core elements in practice. Table 2 presents the subthemes and the NPT construct they relate to. The four themes constitute the structure of the results section.

**Table 2.** Overview of factors identified in the analysis. “+” marks the factors that we identified as promoting the uptake of the core elements, “÷” marks the hindering factors.

	Sickness absence management	Sickness absence prevention
Coherence	Uniform procedures not a new practice	Difficult to decipher old and new practices Lack of shared understanding about preventive actions at the workplaces
Cognitive participation	Procedures are perceived as valuable Procedures ensure a shared managerial focus on absenteeism Procedures perceived as rigid Procedures are not always meaningful or appropriate	-
Collective action	Following the procedures is a requirement Sickness absence management is deprioritized due to busyness. Following the procedures does not make sense.	Lack of shared understanding that sickness absence is a shared responsibility in the workplace Line managers often carry the full responsibility (Experienced) lack of competences Line managers’ own well-being is overlooked Lack of time for intervention activities
Reflexive monitoring	Procedures create structure and clarity about rules and expectations during sick leave. Documentation is cumbersome The widespread desire to be able to deviate from the procedures	Sickness absence prevention primarily focuses on absent employees It is important to also remember the long-term healthy employees Improving the work environment is a more sustainable solution

### Reflexivity

In qualitative research, it is important to recognize how our approach to the research subject, study design, data analysis, and interpretation of results are shaped by our background, research context, and organizational setting (Malterud, 2016). In this study, all authors are affiliated with a research institution dedicated to producing research that supports healthy and safe work environments in organizations. Consequently, we find it essential to consider the work environment as a central factor in addressing sickness absence, which likely influence our approach to the research subject and data collection, data analysis and interpretation of the results.

### Results

The data analysis revealed line manager perspectives on *sickness absence management* and *sickness absence prevention* that relate to all four generative NPT constructs, with the majority relating to collective action (operationalization of a practice). The only exception is that we did not identify perspectives related to *sickness absence prevention* and *cognitive participation*. Table II provides an overview of the points identified in the analysis.

### Coherence

The first construct of NPT is *coherence*, which encompasses how actors understand and make sense of the intervention, e.g., if there is a shared understanding of the intervention in the organization. It also entails whether actors can differentiate the intervention from other efforts and if the intervention is aligned with organizational goals (May et al., 2009). In this study, coherence is the NPT construct that was not particularly evident in the analysis. This could be because neither *sickness absence management* nor *sickness absence prevention* led to significant (if any) changes in how sickness absence is handled. This means that it might be difficult for the line managers to distinguish new practices from old ones, and it can be argued that on this point, the line managers felt that they continued existing practices.

For *sickness absence management*, we found that most of the line managers reported that having uniform procedures for managing sickness absence was not a new practice. According to the line managers, organizational sickness absence policies were already in place before the intervention period, dictating how to report sick from work, how often to contact sick-listed employees, and when to invite an employee with excessive or worrying absenteeism for a sickness absence review meeting. As for *sickness absence prevention*, our findings indicate that the line managers often found it challenging to tell which preventive actions were already in place before the intervention and which actions were introduced with the intervention. In two workplaces, the line managers disagreed about which preventive actions were being implemented in their organization. Some even expressed that no preventive actions were taken besides following the sickness absence procedures and conducting employee development interviews, which are mandatory in Danish public workplaces, and these were already in place before the intervention. Thus, our analysis indicates a lack of coherence for both core elements, possibly due to the line managers not feeling that changes have occurred in how sickness absence is managed.

### Cognitive participation

*Cognitive participation* refers to the work done to engage actors in enacting a new practice, e.g., to ensure commitment and support (May et al., 2009). Above, we argue that the intervention did not result in noticeable changes for the line managers regarding the two core elements. Thus, we cannot examine cognitive participation in terms of the implementation of these. As the line managers referred to already existing practices, we instead investigated motivational factors for adherence to the uniform procedures and for initiating and participating in preventive actions to reduce sickness absence.

*Sickness absence management* is a tertiary prevention strategy, as it dictates formal actions from the line manager towards employees on sick leave. When asked about their opinions on this topic, there seemed to be a shared understanding that having uniform procedures for handling sickness absence was valuable because it ensures follow-up on absent employees. For example, some line managers mentioned that it is easy to forget to follow up on sickness absence on a busy workday, suggesting that the procedures serve as a supportive reminder to contact sick-listed employees. Additionally, some of the line managers think that non-adherence to the procedures would prolong the duration of the absenteeism:

It is important to stay in touch with your workplace, and the sick leave does not become long. The longer the sick leave, the further away you are from the labor market.

According to the line managers, the procedures ensure early contact with the sick-listed employee, influencing the employee’s chances of returning to work. They also mentioned that uniform procedures ensure that all line managers focus on managing sickness absences:

I believe that as line managers, we are very different. Some have much focus on absenteeism, the work environment, and well-being, while others have a different or less focus on it. In that light, it is good that we have these procedures.

At the same time, some of the line managers felt that the procedures were too rigid and deprioritized the line managers’ own opinions and experiences even though they knew their employees best. In line with this, some of the line managers reported that there are instances where uniform procedures are not appropriate. For example, if an employee has been terminated and subsequently takes sick leave in the remaining period of employment (in Denmark, there is usually a notice period that can last for several months, depending on the length of employment), the procedures were not perceived as meaningful. A line manager elaborated:

If an employee has been terminated, I must admit that I do not call them. It is a waste of time. Then I can call and ask if the sun is shining. That would also be stepping on the employee’s toes a bit. There is no reason for it. The contact has to be meaningful.

As the quote indicates, making contact alone is not enough. There has to be a meaningful purpose for contacting absent employees (i.e., establishing how the line manager can support the employee’s RTW). Otherwise, it is perceived as unnecessary and inappropriate for the employee.

Some of the line managers mentioned that there is a difference in whether an employee is absent due to physical or mental reasons and that it must be managed differently. These examples suggest that it may not always make sense for line managers to apply a uniform model in handling all types of sick leave.

As stated, we found no promoting or inhibiting factors relating to sickness absence prevention and cognitive participation, and this is likely due to a lack of knowledge or transparency about preventive measures taken in the organization.

### Collective action

“Collective action” refers to the work that people do, individually or collectively, to enact a new practice (C. R. May et al., 2009). In our analysis, collective action, in relation to *sickness absence management*, focuses on the line managers’ adherence to the sickness absence procedures in practice and the factors that hinder or promote adherence.

All line managers expressed that they follow the procedures most of the time. However, some line managers mentioned that they primarily follow the procedures because it is a requirement dictated by the organizational policy on sickness absence, and because the HR department follows up on whether the line manager contacts sick-listed employees or conducts sickness absence review meetings in accordance with the procedures:

We do not have much choice. We have to follow the procedures dictated by [the top management]. I may have my opinions about it, but it is something I have to do.

Of course, it is part of overseeing that I, as a manager, am attentive to the department I am responsible for, and that I handle sick leave according to the requirements and regulations.

Specifically for the ongoing contact with employees on sick leave, the original purpose was for the line manager and the employee to collaboratively determine the best way for the employee to RTW. In several interviews, it is mentioned that this contact is a way for the line manager to demonstrate care and interest in their employees. However, some line managers see this contact as a mandatory managerial task, a perception that the line managers also believe is reflected in the employees, indicating that the care and interest may not be perceived as sincere. Showing genuine interest is otherwise highlighted as necessary for how the employees experience being contacted during sick leave:

If we tell the employee that we are calling because we have to, we lose both ourselves and the employees. There must be a genuine interest in the well-being of our employees. If that is what we stand for, consistently, even in day-to-day interactions. Well, they have always appreciated my calls.

The line managers also gave examples of non-adherence to the procedures. This was often related to busyness, where contacting sick-listed employees was deprioritized compared to other work tasks. One line manager mentioned a challenge in wholeheartedly managing the contact because it had to be squeezed between other meetings and tasks:

The workload is heavy, and sometimes, you just breeze through the sick leave interview because you know that in about 20 minutes, you have the next [meeting or conversation].

Another line manager mentioned a situation where following the procedures did not make sense, resulting in following another approach:

We have had two serious cases of cancer. We handle those differently. The obvious cases where it does not make sense because we all know nothing has changed from day 1 to day 5. In those situations, we make other arrangements.

For *sickness absence prevention*, collective action refers to factors that affect the implementation of preventive actions in their departments. Several line managers highlight the importance of establishing a common understanding that sickness absence is a shared responsibility that requires commitment from all levels in the organization. However, some of the line managers often find themselves shouldering the full responsibility for managing sickness absence alone. The line managers experienced that employees tended to push off the responsibility, expecting the line managers to handle everything. While the line managers acknowledge their central responsibility in managing sickness absence, they also need the employees to take on more responsibility:

Employees often look to the leadership to solve the problem. While it may be true that the leadership needs to come up with solutions, we also need to involve the employees in the process to make it thrive within the employee group […] But if you have employees thinking it is a management problem - it is not! It is a management problem, but it is also the employees’ problem.’

Furthermore, some of the line managers did not feel adequately equipped to handle sick leave. It is articulated that they lack competencies and knowledge, especially in complex cases. For example, a line manager who has employees on sick leave due to post-traumatic stress disorder (PTSD), mentioned that a lack of knowledge about how the symptoms of PTSD are expressed was an obstacle to being able to handle the absence most appropriately. The line manager further explained:

I am the only one talking to [the sick-listed employees], but I am not properly prepared. I try to show them compassion, understand their situation, and show empathy, but it is on a human level. We can read up on procedures and everything, but we have many employees dealing with PTSD, and we have no knowledge about it.

While it cannot be expected that line managers must possess in-depth knowledge of mental health problems, their perspectives are about understanding how to support employees experiencing these problems. To do so, the line managers expressed a desire for better opportunities for seeking information, knowledge sharing, and collaboration with other line managers.

Some of the line managers also report that their own well-being is overlooked in relation to sickness absence. The line managers often have to deal with employees’ illnesses and personal problems but do not have a place where they can ventilate:

We focus so much on preventing employee absenteeism, but what about the leadership aspect? That is often overlooked, I think. I have no one to turn to. How do you keep finding energy and resilience when dealing with all that illness? Because there is a lot of illness when you are in charge of a large department.

In addition, the heavy workload experienced by the line managers may result in exhaustion and not being able to be present for the employees:

If you do not have good leadership, if you have a tired leadership, it reflects in the employees. You cannot give something you do not have yourself. How do we focus on ensuring the well-being of [the line managers]?

We are getting more and more work tasks, but we are not getting more time. The more absenteeism among our employees, the more burdensome it is to be a leader, and the harder it becomes to keep smiling and be friendly.

Lastly, the line managers mentioned that a lack of staff and economic resources, increased demands, additional work tasks, and time pressure limited the time needed to implement preventive actions. According to the line managers, it is not possible to allocate time for initiating preventive actions, such as pulling employees out of their regular tasks to participate in workshops or training, because it is necessary to focus on ensuring daily operations.

### Reflexive monitoring

*Reflexive monitoring* includes formal or informal assessment of a new practice, e.g., whether it is perceived as advantageous or if it can be adapted or improved to enhance integration (C. R. May et al., 2009). In this study, reflexive monitoring focuses on the line managers’ assessment of both core elements and which adjustments they suggest to enhance their effectiveness in practice.

Regarding *sickness absence management*, the line managers expressed that the procedures for reporting sick from work are well-known among the employees, meaning that their employees know whom to contact when feeling too ill to attend work. The line managers themselves thought that the procedures provided a more transparent structure for how sickness absence is handled and facilitated a more coherent understanding of how sickness absence is managed in the workplace by providing a clear alignment of expectations for both managers and employees:

The structure has actually improved. Both for the employees - it is more transparent now what we expect from them and when they are scheduled for a meeting. It is also better for us as managers who handle. [the sick leave]

Furthermore, the line managers expressed that the procedures ensure equal treatment of the employees and that they support them because they establish legitimacy and clarity about the rules and the purpose of the procedures. A line manager elaborates:

There is a legitimacy in it when one knows that is how the rules are. When employees are aware that [we call them] on the first day, everyone is familiar with the fact that it is not something the manager does to be annoying; it is to ensure that we get the employees back on their feet as quickly as possible.

When asked how the line managers think employees experience being contacted during sick leave, they shared mixed experiences with the ongoing contact. While some line managers expressed positive employee attitudes towards the contact, other line managers mentioned that their employees tended to perceive the contact as controlling:

I think employees may perceive it as control. Some employees are more affected by it than others, and some can take it very personally.

Moreover, some of the line managers were somewhat frustrated about having to document adherence to the procedures. In practice, the line managers must document that they contacted the employee and what they discussed. The line managers describe the documentation as a heavy work task:

These rules and guidelines are perfectly fine, but it is administratively heavy to have to document, document, document.

Other frustrations revolve around the perception that documenting is cumbersome because the line manager must use different administrative systems to do so or that the documentation is not utilized afterwards. Furthermore, a line manager articulates that there is greater emphasis on documenting adherence to the procedures rather than focusing on the importance and value of the dialogue between the line manager and the employee.

I feel that the need to document [adherence to the procedures] is almost greater than the need for and the importance of why we [contact the employees]. I believe having contact with the employees and following them closely is crucial. I find that more important than documenting it.

Despite the negative aspects, the line managers generally perceived the uniform procedures as an integrated part of managing sickness absence and were primarily positive towards their effects. However, some suggestions for alterations were made. For example, in all four workplaces, the procedures included contact between the absent employee and the line manager on the first day of the sick leave. This did not make sense for the line managers, as many employees typically return to work after a few days. Therefore, several line managers would like to deviate from contacting employees already on day 1.

For *sickness absence prevention*, the line managers mentioned that the preventive actions in their workplaces were reactive, only focusing on employees already on sick leave (tertiary prevention). Several line managers expressed a need for more proactive, primary actions to prevent sickness absence from occurring in the first place:

Could it have been avoided if we had cared more about our employees? If we had acknowledged them more? If we had invested more in them? I am sure it could have.

Furthermore, some of the line managers thought that too much emphasis is put on absenteeism. Specifically, they mention how it can negatively affect employees who are rarely absent to be reminded about the high incidence of sickness absence constantly. There should also be a focus on recognizing the remaining employees who often carry the workload when their colleagues are absent. Additionally, several line managers addressed the risk of co-workers burning out when covering for a sick-listed colleague, but this is not taken into account in the preventive actions:

We put so much focus on those who are sick. But what about all those who are actually doing their job well, showing up, and keeping operations running?

A line manager mentioned that despite challenges with absenteeism, many employees are rarely absent. According to some of the line managers, these employees expressed frustration continuously hearing about absenteeism, suggesting that it is essential, as line managers, to consider how to communicate about absenteeism to the employees, shifting focus to the healthy ones as well.

In line with this, when we asked the line managers what they believed would help prevent sickness absence, they highlighted the greater importance of ensuring well-being and job satisfaction among the employees (primary and secondary prevention) rather than focusing on absenteeism (tertiary prevention):

It does not lead to increased employee well-being or job satisfaction when we focus on absenteeism, scheduling meetings here and there, and calling people. That is not what makes us a good workplace.

To summarize, although the line managers were generally positive about having specific frameworks for managing sickness absence and acknowledged the importance of ongoing contact with sick-listed employees to support their RTW (primary prevention), there was a widespread desire for more focus on creating a good work environment and fostering well-being (primary and secondary prevention), as it was considered a more long-term and sustainable solution.

### Discussion

This study examined line manager perspectives on *sickness absence management* and *sickness absence prevention*, two core elements from a complex intervention framework aimed at reducing sickness absence. The four NPT constructs (coherence, cognitive participation, collective action, and reflexive monitoring) inspired the analysis.

Although the two core elements were included as part of the intervention, the results indicate that either significant changes regarding sick leave procedures or sick leave prevention have not occurred or that participants could not distinguish between new and old practices. However, the results revealed a series of points of attention that should be considered if 1) the two core elements are to function as intended, and 2) the intervention as a whole is to be successfully implemented, thereby achieving an effect on sickness absence.

The first point we want to address is that we found widespread positive cognitive participation regarding *sickness absence management*, where the line managers expressed a shared understanding that it is valuable to have a framework for managing sickness absence. Previous research argues that workplace contact during sick leave is essential (Buys et al., 2019), which was also expressed by the line managers in this study. However, as seen in the section “reflexive monitoring”, the line managers mentioned that making contact on the first day (referred to as “early contact”) and following a rigid structure did not always make sense. A systematic review found that early contact between an absent employee and the workplace could reduce work disability duration (Higgins et al., 2012). On the other hand, Selander and colleagues investigated the pattern and quality of workplace contact for employees on long-term sick leave. They found that what mattered more in relation to return to work was the quality of the contact rather than the frequency of the contact and how early in the sick leave it is initiated (Selander et al., 2015). This finding is supported by Tjulin et al. (2011) who argue that early contact might not always be the best approach and that it is necessary to consider individual needs (Tjulin et al., 2011). Some of the line managers in this study did not find it meaningful to use the same model for all employees. This suggests that the line managers perceive it more appropriate to tailor the ongoing contact to the employees’ needs. This indicates that more individualized planning can enhance the quality of the contact. Furthermore, the results show that the line managers in practice perceive the procedures for handling sickness absence in such a way that there is a greater emphasis on being able to prove adherence to the procedures than on the actual purpose of the procedures, which is to support the RTW process of absent employees.

The focus of the organizational policy on ongoing contact suggests that the primary aim of the procedures is to support the absent employee’s RTW (tertiary prevention). While most of the line managers acknowledge this value, some also expressed a need for proactive actions that prevent sickness absence from occurring in the first place (primary and secondary prevention). When examining perceptions of *sickness absence prevention* (addressing challenges in the psychosocial or physical environment), line managers expressed a desire for a greater focus on especially primary prevention of sickness absence, and some line managers highlighted that it would be valuable to ensure well-being and job satisfaction instead of solely focusing on absenteeism. Previous research has demonstrated that risk factors in the work environment, such as high emotional demands, low influence on the job, role conflict, and violence and threats, are associated with sickness absence and that interventions aimed at improving the work environment could have a positive impact on sickness absence (Rugulies et al., 2007; Sundstrup et al., 2018). However, in our study, most of the preventive actions highlighted by the line managers were not directly aimed at the work environment and were, according to the line managers, not initiatives but mandatory activities, such as Employee Development interviews (a dialogue between the line manager and employee about, for example, job satisfaction, well-being, and professional and personal development). Our findings indicate that sickness absence prevention lacks operationalization across the four workplaces or that there is a lack of communication or transparency in the organization about which preventive actions that are being implemented. Generally, our findings indicate that even though the core element suggests that the work environment should be considered to prevent sickness absence (primary and secondary prevention), this does not happen in practice. Instead, there is a greater focus on tertiary prevention.

In line with this, the body of literature on sickness absence interventions often focuses on employees with mental health problems, chronic illness, or musculoskeletal disorders (Axén et al., 2020; Nigatu et al., 2016; Odeen et al., 2013), i.e., employees already on or at risk of sick leave. The line managers in this study reported that too much focus is put on absenteeism, and that it is also essential to consider how it affects the remaining colleagues, who not only have to cover for the sick-listed employee but are also at risk of sick leave themselves. This indicates that future interventions could advantageously be more proactive and focus on the primary prevention of sickness absence by aiming to improve the work environment for all employees rather than focusing on getting already sick-listed employees back to work as quickly as possible.

As part of the “collective action” theme, it was mentioned that it could be challenging to find the energy to manage sickness absence wholeheartedly. This was partly due to the burden of dealing with others’ illnesses and problems and partly because the line manager must handle increasing tasks without being allocated additional time. The line managers expressed that their own well-being was often overlooked in favour of the employees. This finding is interesting, as we have not come across studies that address the well-being of line managers and its impact on handling employees’ sickness absence. However, the well-being of line managers is essential to consider. A study on line manager resilience (Coutinho & Carder, 2022) argues that line managers whose health is affected by work may be less capable of ensuring the well-being of their employees, a point also expressed by the line managers in this study. It was mentioned that a stressed line manager might not be able to exhibit the same resilience towards their employees, which can be problematic considering that studies have argued that the quality of line manager interaction (focusing on care, trust, and respect) may affect employee well-being (Renee Baptiste, 2008; Skakon et al., 2010).

Furthermore, the central role of line managers in relation to sickness absence has been highlighted. For example, a paper by Abma et al. (2013) describes line managers as key figures “*in the work functioning of workers with health problems*” (Abma et al., 2013, p. 20), an argument supported by Preece, who argues that the behaviour of line managers affects whether an employee resumes work successfully following sickness absence (Preece, 2019). This suggests that future interventions should consider line managers’ well-being as well.

Moreover, the line managers reported lacking competencies to handle sickness absence appropriately. This was also found in another study investigating barriers and facilitators for thriving at work for employees post sick leave (Nielsen & Yarker, 2024), indicating the need for employers to provide training to line managers with staff responsibility on how to communicate with and support employees during and after sick leave. This is in line with other studies that argued for the importance of providing training on, for example, communication skills and facilitating workplace accommodation to enhance return to work (Buys et al., 2019).

### Implications

The study findings give rise to some practical implications for organizations (see Table 3). These implications can contribute to making the prevention and management of sickness absence more tangible and meaningful for line managers. Arguably, addressing some of the challenges presented in this study can make the prevention and management of sickness absence more effective, thereby reducing sickness absence.

**Table 3.** Key practical implications from the findings.

Key practical implications	
1	Procedures for the management of sickness absence could be made more flexible to allow the line manager to take individual considerations into account, such as the employee’s situation, needs and work function.
2	Workplaces should consider how leaders can be supported in challenging cases with employees on sick leave, such as ensuring adequate training or access to relevant knowledge and resources.
3	Clear communication regarding proactive, primary preventive measures to improve the work environment and well-being, and the connection between these measures and the prevention of sickness absence is needed.
4	Involving line managers in shaping an organization’s sickness absence policies and absence management practices may likely enhance the operationalization and effectiveness of these in practice.

In terms of *sickness absence management*, workplaces should allow the organizational procedure on sickness absence to be more flexible to allow the line manager to take individual considerations into account, such as the employee’s situation, needs and work function. If the procedure is already flexible, workplaces must ensure transparent communication with line managers regarding the framework for considering individual factors in communication with sick employees is necessary. The requirements for documentation should also be considered, if possible, to minimize the burden. Alternatively, workplaces must consider how to make documentation easier for the line managers, such as ensuring that they only need to use one system to do so, or make sure that the documentation is utilized afterwards to make it more meaningful for the line managers to spend time doing it.

Moreover, workplaces should consider how line managers can be supported in challenging cases with employees on sick leave. The workplace can, for example, support the line manager by sparring with managerial colleagues and training communicative skills. The workplace may also have guidelines for adapting work to increase the chances of a successful RTW. It should also be considered to strengthen the line manager’s access to knowledge about how various health problems manifest themselves and affect the ability to work, especially for the health problems that most often cause sick leave at the workplace. This could, for example, be made available through the intranet.

Regarding *sickness absence prevention*, the findings indicate a lack of cognitive connection between sick leave and primary prevention (such as improving well-being and the work environment). This should be viewed in conjunction with some line managers’ perception that sickness absence receives too much attention, overshadowing the many employees who are never or rarely absent. Therefore, workplaces should ensure clearer communication regarding primary preventive measures to improve the work environment and well-being for all employees, as well as the connection between these measures and the prevention of sickness absence.

Furthermore, the results underscore the importance of involving line managers in shaping an organization’s sickness absence policies and absence management practices. One aspect is having such policies and procedures, while another is whether and how they are implemented in practice. This aspect—how policies and procedures are operationalized—is often overlooked. Line managers are the ones who work with the policies and procedures in practice, and thus have insight into what works and what does not. Additionally, line managers understand their employees and departments best, including what procedures and preventive actions that might work best for them. Involving line managers and their experiences in developing and implementing procedures and preventive measures is likely to enhance operationalization and effectiveness of these in practice.

Additionally, this study adds to the body of literature on occupational health interventions, as it emphasizes the importance of considering the work environment to ensure healthy and safe working lives for employees when making efforts to reduce sickness absence. As argued, there are several risk factors for sickness absence in the work environment, and it has been argued that improving the work environment has the potential to reduce absenteeism by up to 30 % (Mathisen et al., 2022), underscoring the importance of primary and secondary prevention strategies. Finally, the study emphasizes that workplaces should be mindful of the central role of line managers in managing sick absence and how they can be supported in terms of well-being and competencies, to ensure that they are equipped to support the well-being of employees. For example, workplaces can be attentive to line managers’ well-being by including this aspect in their workplace risk assessments or well-being surveys, and based on this, develop action plans to support the line managers. Another suggestion is to establish leadership networks where line managers can express their thoughts and experiences in a confidential forum with other line managers.

## Methodological considerations

The study complements existing research on the role of line managers in the prevention and management of sickness absence. Overall, there are several overlaps between the study’s results and existing knowledge, but the study distinguishes itself by highlighting the importance of primary prevention of sickness absence, even though the results indicate that, in practice, there is a much greater focus on tertiary prevention of sickness absence (RTW processes). Another contribution is the importance of being mindful of line managers’ well-being, as it can impact their ability to ensure well-being and thereby reduce sickness absence among their employees. Furthermore, the study contributes with knowledge about the factors that affect effective prevention and management of sickness absence, including influential factors affecting the central role of the line manager and what they do in practice regarding sickness absence.

However, there are some methodological considerations that we need to address. Firstly, we used an implementation theory as theoretical framework even though implementation was not our direct focus for this study. Our initial concern with using a predetermined theoretical framework was that we might force our data into rigid categories. However, the authors of NPT have argued that the theory should be seen as a flexible reference framework; thus, there is no single prescribed way to apply NPT (C. R. May et al., 2018). In our experience, it was significantly helpful to start the analysis process with inductive coding and then use NPT as a flexible lens to illuminate our data rather than as rigid categories. Using this approach, NPT was a helpful framework for identifying potential, influential factors for operationalizing the two core elements in practice. However, overlaps between the constructs made the analysis difficult. There were considerable overlaps between cognitive participation and reflexive monitoring, as it was sometimes difficult to tell whether the perceptions expressed by the line managers related to motivational factors for the uptake of or assessing the value of the core elements. Overlaps were also challenging in other NPT studies (McEvoy et al., 2014).

Nevertheless, using NPT as a theoretical framework strengthened the analysis. NPT has contributed to identifying underlying challenges for making the prevention and management of sickness absence feel meaningful for the line managers (coherence), for ensuring that line managers are motivated to actively engage in the prevention and management of sickness absence (cognitive participation), for operationalizing activities related to prevention and management in practice (collective action), and for determining what changes are needed for better and more appropriate prevention and management of sickness absence (reflexive monitoring). In this way, the theory has helped us identify key promoting and hindering factors for an effective prevention and management of sickness absence that can be transformed into practice-oriented knowledge.

Furthermore, the need for theory to explain social processes during implementation has been highlighted in previous papers (May, 2013). While other studies have examined line manager attitudes in relation to sickness absence, our study differs in that we apply a theoretical framework. For example, Ladegaard et al., Norvell Gustavsson et al., and Wynne-Jones et al. all examined line manager attitudes or actions towards employees on sick leave or during the RTW process but did not use any theory to explain or illuminate their findings (Ladegaard et al., 2019; Norvell Gustavsson et al., 2021; Wynne-Jones et al., 2011).

Secondly, although *sickness absence management* and *sickness absence prevention* were described as two different intervention components, and we treated them accordingly, most of the line managers tended to perceive *sickness absence management* as a part of *sickness absence prevention*. Therefore, they often regarded the two core elements as one. For this reason, it was not always possible for us to decipher whether the perceptions articulated by the line managers were related to *sickness absence management* or *sickness absence prevention*. Therefore, they were in our analysis at places treated as one component.

Furthermore, data collection took place during the COVID-19 pandemic. Implementing preventive actions during a crisis, where both line managers and employees had to work under particular conditions and adhere to restrictions, significantly limited the possibility of prevention. In the four workplaces participating in this study, most employees continued to meet physically at work. Thus, the pandemic did not have significant consequences regarding contact between line managers and employees. However, the workplaces were, just like everyone else, affected by the pandemic in the form of increased infection pressure and the introduction of new procedures to prevent the spread of infection in the workplace. However, it is unclear to what extent these precautions have influenced the results of this study.

Furthermore, coding was done by the first author alone. Measuring intercoder reliability is usually considered good practice in qualitative research, but this was not possible in our study. However, the authors of TA argue that intercoder reliability is not necessary. On the contrary, it is argued that having only one coder is a common and sound practice in TA, as researcher subjectivity should be seen as a strength rather than a weakness (Braun & Clarke, 2021). Nevertheless, this remains an interesting methodological consideration.

Regarding strengths of this study, we created important new knowledge about how workplaces can enhance primary and secondary prevention of sickness absence. This is an important contribution to research and practice, because (as presented in this paper) previous research and practice have focused on tertiary prevention strategies but this paper has pointed to the importance of also applying primary and secondary prevention strategies, as this is considered a more sustainable solution to the complex challenge of reducing sickness absence.

The number of participating line managers and the purposeful sampling strategy is also a strength because it allowed us to include line managers from different levels and work areas in the public sector, from departments of various sizes and with different seniority, meaning that we could explore the research focus from several perspectives. This provided us with a nuanced data set based on a broad section of line managers in the Danish public sector. In Denmark, most public sector workplaces have organizational policies on sickness absence, suggesting that these findings are relevant to other work areas than the ones included in this study. However, transferability to workplaces in the private sector or small and medium enterprises must be done carefully, as they may have other approaches to management and prevention of sickness absence.

The limitation of the study is that we did not include interviews with employees to examine their perspectives on the two core elements. This would have provided a more holistic understanding of the efforts examined in this study. Future research on sickness absence management and prevention should include employee experiences as well.

## Conclusion

The results highlight the importance of a structured framework for managing sickness absence that clarifies roles, responsibilities and rules for both line managers and employees. Line managers in the study expressed a need for more flexibility in the application of procedures for contacting employees on sick leave to meet the needs of individual employees. They stressed that workplace contact must be meaningful to be effective, and not just a formal requirement that simply has to be met. While measures to support employees’ RTW (tertiary prevention) were well established in the organizations and among line managers, line managers identified a lack of efforts to prevent sickness absence at an earlier level, i.e., primary and secondary prevention measures. Line managers also experienced a lack of knowledge and skills to manage absence when the causes of sickness absence are complex. Furthermore, they noted that an important prerequisite for being able to support employees is that their own well-being is good, which was often overlooked. These results suggest that sickness absence management should have an increased focus on primary and secondary preventive measures and support for managerial well-being. Future research could explore interventions that focus on strengthening these aspects of sickness absence prevention.

## Data Availability

Data is available (in Danish) upon reasonable request.
